# When emotion meets reason: the development and validation of EpiCT-CI scale to measure epistemic emotions in critical thinking application and cultural identity constructions

**DOI:** 10.3389/fpsyg.2025.1687003

**Published:** 2025-10-15

**Authors:** Yue Peng

**Affiliations:** General Education Centre, Guangzhou Academy of Fine Arts, Guangzhou, China

**Keywords:** epistemic emotion, critical thinking, cultural identity, scale development, cultures

## Abstract

**Introduction:**

Epistemic emotion is a significant concept in education, but traditional scales rarely focus on the status of epistemic emotions in intercultural issues. Additionally, cultural identity and critical thinking are vital in navigating the complexities inherent in intercultural contexts. Existing measures of critical thinking and cultural identity seldom consider the influence of emotions. The EpiCT-CI Scale, developed in this research, seeks to bridge this gap by measuring how epistemic emotions influence critical thinking and cultural identity in intercultural settings.

**Method:**

Developing and validating the EpiCT-CI Scale combines qualitative and quantitative methods. Study 1 collected data from students’ comments, judgments, and narrations about critical thinking during COVID-19. Study 2 focused on the emotional experiences of constructing cultural identity by reading, analyzing, and writing about cultural issues. The data from Studies 1 and 2 are analyzed in NVivo 15.0. The original EpiCT-CI Scale is validated through SPSS 20.0 and Amos 29.0 in Study 3.

**Results:**

The results from Studies 1 and 2 indicate that epistemic emotions are a blend of neutral, positive, and negative states, rather than simple linear progressions. The initial 52-item scale underwent a thorough evaluation, modification, and validation process in Study 3, resulting in a four-dimensional 19-item EpiCT-CI Scale, which represents four groups of epistemic emotions: joy in critical cultural inquiry, boredom in critical cultural reflection, curiosity in cultural identity reflection, and distress in cultural adaptation. The EpiCT-CI Scale provides an effective tool for assessing epistemic emotions in cultural identity constructions and critical thinking applications.

## 1 Introduction

Emotion is generated by understanding experiences, beliefs, values, and imagination. It is not merely a physical or psychological phenomenon but is closely related to the cultural background and is profoundly influenced by cultural rules, language, and social practices (Boellstorff and Lindquist, 2004; Kotchemidova, 2010). Epistemic emotions can be considered as emotions about learning, which play a critical role in regulating how people engage with information, especially when encountering cognitive dissonance or uncertainty (Pekrun et al., 2017). When dealing with cultural challenges, epistemic emotion encompasses the features of emotions, but in a more cognitive way. When addressing cultural diversity, epistemic emotion, cultural identity, and critical thinking are interconnected. Integrating epistemic emotions, critical thinking, and cultural identity development would be a crucial approach for understanding how university students can survive and thrive in the complexities of cultural diversity. This research aims to develop a measurement to investigate the status of epistemic emotions. The EpiCT-CI Scale can measure art students’ status of epistemic emotion when actively applying critical thinking to cultural identity constructions in challenging cultural issues. It provides a valuable tool to assess how students manage their epistemic emotional responses when engaging in multicultural situations.

## 2 Literature review

### 2.1 The epistemic emotion and cultural identity

Epistemic emotions can be defined as emotions “that are caused by cognitive qualities of task information and the processing of that information” (Muis et al., 2015b), which arise when individuals focus on knowledge and knowing (Muis et al., 2015a). Emotions are a wide range of physical and psychological phenomena. Scarantino (2025) divides the emotions into eight groups: “protecting the body (e.g., pain, fear), improving decision-making and goal achievement (e.g., desire, stress, surprise), fostering skills development (e.g., amusement, interest), improving communal living and interpersonal relations (e.g., guilt, empathy, gratitude), creating and upholding systems of norms (e.g., embarrassment), moving within status/positional hierarchies (e.g., envy, pride), contributing directly to wellbeing (e.g., pleasure, hope), and procreating and caring for the offspring (e.g., love, compassion).” According to Muis et al. (2015b), epistemic emotions mainly “include, but are not limited to, surprise, curiosity, enjoyment, confusion, anxiety, frustration, and boredom.” Besides, Pekrun et al. (2017) propose the Epistemically related Emotion Scales (EES) to outline the other 16 specific epistemic emotions, which include interested, anxious, inquisitive, dull, amazed, worried, happy, muddled, irritated, monotonous, excited, astonished, nervous, joyful, and puzzled. These emotions are related to the degree of conflicts, challenges, and puzzles in “acquiring knowledge about the world and the self (Pekrun et al., 2017).”

Epistemic emotions can be considered multi-dimensional constructions. Based on the Control-Value Theory (CVT, Pekrun, 2006), the epistemic beliefs and self-regulated learning model (Muis, 2007), and the integrated model of epistemic beliefs (Bendixen and Rule, 2004), Muis et al. (2015b) propose the integrative model of epistemic beliefs, epistemic emotions, and learning. This model suggests that epistemic beliefs influence the generation of epistemic emotions, affecting learning strategies and outcomes. Muis et al. (2018) further indicate that epistemic emotions in self-regulated learning can be aroused by five factors: “control, value, novelty, complexity of information, and the achievement or impasse of an epistemic aim.” When engaging in learning, the individual’s perceptions of the task, information, and target are significant for epistemic emotions. The perception of control and value can be considered as a sense of task assessment that predicts the various epistemic emotions, such as joy, anxiety, or boredom; the perception of novelty and information complexity can be viewed as the information evaluation that triggers the surprise, curiosity or confusion; the perception of achievement or impasse of an epistemic aim predicts enjoyment or frustration.

Emotions are crucial in forming and developing identity; identity also significantly influences emotional responses. Identity is an active, self-constructed “being” process that can vary depending on individual perspectives (Berzonsky and Papini, 2014; Berzonsky, 2016; Berzonsky and Kinney, 2019). A strong commitment to one’s identity can enhance a sense of pride, but it may also result in anxiety when expectations are not fulfilled (Mackenzie, 2002). Identity is a “creation” that involves a process of self-driven actions (Berzonsky, 2016). Positive psychology views identity construction as a developmental process that involves behaviors such as exploration, reflection, and negotiation. Waterman (2015) summarizes identity development into four dimensions: “(a) exploration in breadth, (b) exploration in depth, (c) reconsideration of commitment, and (d) ruminative exploration.” In this process, individuals experience varying degrees of emotional fluctuation. The research focuses on the relationship between emotion and identity and mainly covers the different groups of people in intercultural contexts (Abbott and Burkitt, 2023; Gürsoy, 2023; Derakhshan et al., 2023; Karimpour et al., 2024; Yoshida, 2024) and educational settings (Arslan, 2023; Kettunen et al., 2023; Yazan, 2023; Kang, 2024; Fisher et al., 2024; Wang et al., 2021). These studies indicate that identity construction involves the multi-dimensional ongoing process by which individuals form and refine their sense of self, including emotions, social roles, and beliefs.

Epistemic emotions and cultural identity are interconnected. Jameson (2007) defines cultural identity as “an individual’s sense of self derived from formal or informal membership in groups that transmit and inculcate knowledge, beliefs, values, attitudes, traditions, and ways of life.” The cultures, the changing nature of identity, and the different emotional expressions work together to form our unique story of cultural identity. Cultures have a multifaceted impact on emotion, intertwined with emotional recognition and expression, positive and negative emotions, cultural values and religious beliefs, or economic and political factors (Van Hemert et al., 2007). Boellstorff and Lindquist (2004) use the example of “shame” in Southeast Asian culture to illustrate the impact of the culture on individual emotional experiences and cultural identity. Epistemic emotions can influence an individual’s identity by affecting how they process and integrate new knowledge into their self-concept, and cultural identity also influences the pattern of epistemic emotions. Some studies indicate a complex interplay between epistemic emotions and cultural identity, such as the diverse elements influence the individuals’ epistemic beliefs and emotions in cultural and intercultural practices (Gottlieb, 2007; Odebiyi and Choi, 2022); or the construction of cultural identity also shapes the evaluation of knowledge (Mato, 1996; Tisdell, 2006; Bortolan, 2024; Padilla Cruz, 2024). Given the significant impact of epistemic emotions on cultural identity, it is essential to integrate critical thinking in addressing the challenges and difficulties that arise from cultural diversity.

### 2.2 The epistemic emotion, critical thinking, and cultural identity constructions

Critical thinking involves emotions in many ways, for it cannot function completely rationally. Critical thinking is the skill to observe, forecast, analyze, evaluate, infer, reflect, and reason to solve problems. It is purposeful and consists of solving problems, formulating inferences, calculating probabilities, and making decisions (Halpern, 2014). Stanovich and West (2000) claim that the thinking patterns of System 1 (emotional, fast, intuitive thinking) and System 2 (analytical, slow, critical thinking) are cooperating to work. When dealing with problems, a critical thinker should be one with “critical spiritedness” in mind, such as “love for truth, open-mindedness, fair-mindedness, self-confidence, and intellectual courage to describe what kind of person a critical thinker is” (Pettersson, 2020). Steinert et al. (2025) further state, “A genuine critical thinker is not only open to new empirical evidence but also actively seeks out perspectives that could destabilize their values and norms.” Critical thinking goes beyond mere reasoning and usually causes strong and challenging emotional experiences because being a critical thinker can overrun personal values and goals (Steinert et al., 2025). Emotions influence critical thinking directly or indirectly, for example, by enhancing emotional experiences to promote more profound reflection on complex issues (Bull and De Angeli, 2021), by creating an emotionally supportive environment to promote critical thinking skills (Zhang and Zhang, 2013; Danvers, 2016), or by providing psychological safety for critical thinking application (Candiotto and Slaby, 2022; Christodoulakis et al., 2023).

The interrelations between critical thinking and epistemic emotions are evident in adjusting beliefs. Epistemic emotions and epistemic beliefs are closely related (Pekrun et al., 2017). Pekrun et al. (2017) state that epistemic emotions serve “evolutionary-based purposes of acquiring knowledge about the world and the self” and share the same goal as epistemic belief. The integrative personal epistemology model developed by Bendixen and Rule (2004) involves three parts: epistemic doubt, epistemic volition, and resolution strategies, which explain how individuals create and adjust their epistemic beliefs in different environments and situations. This model offers a more holistic perspective on the elements that evoke epistemic emotions, such as cognitive abilities and cultural contexts. Additionally, Halpern (2014) proposes that developing the disposition for effortful thinking and learning is significant for applying critical thinking. A stronger belief in meaningful learning is associated with the more effective use of learning strategies (Shinogaya, 2008, 2011, 2018). Muis et al. (2015a,b) suggest a positive correlation between epistemic emotions and critical thinking. When engaging in the learning process, the individuals “who believe that knowledge is simple, certain, ……and passively constructed (i.e., less constructivist beliefs), may experience surprise, confusion, anxiety, frustration, and boredom, whereas those who believe that knowledge is complex, uncertain, justified through inquiry and critical thinking (i.e., more constructivist beliefs), may experience curiosity and enjoyment.” The scope of Halpern’s (2014) “disposition” about critical thinking can also be comprehended as the “more constructivist beliefs (Muis et al., 2015a,b), which represent the attitudes and beliefs about the importance of questioning assumptions, the value of diverse perspectives, and the necessity of reflective thinking.

Moreover, the interplays between critical thinking, cultural identity, and epistemic emotions are reflected in information processing. Epistemic emotions emerge from “information-oriented appraisals about the alignment or misalignment between new information and existing beliefs, existing knowledge structures, or recently processed information (Muis et al., 2018).” Cultural identity construction encompasses specific tasks that deal with different cultural information. As Dervin and Yuan (2022) state, cultural identity is “reflecting on themselves, others, and the world while interacting with them. “The epistemic emotions enhance the commitment to cultural identity. Trevors et al. (2016) state that when individuals encounter information that aligns with their cultural identity, they are more likely to experience positive epistemic emotions such as curiosity and interest. However, information contradicting their cultural identity can lead to negative emotions like confusion, anxiety, and frustration. Critical thinking enables individuals to reflect on their cultural affiliations by analyzing, evaluating, or reflecting (Collins, 2018; Sato and Horn, 2023; Peng, 2024). Studies on identity negotiation demonstrate that critical thinking is a transformative tool for overcoming cultural prejudices and systematic imbalances (Caldwell, 2012; Sheybani and Miri, 2019; An Le and Hockey, 2022). Epistemic emotions can either facilitate or hinder critical thinking in constructing cultural identity. Muis et al. (2021) state that confusion and anxiety can be positive predictors of critical thinking, while frustration is a negative predictor that leads to an excessive burden on the cognitive system and reduced effort to apply critical thinking. Thus, critical thinking is significant for helping individuals to manage the complexities of new or conflicting cultural information.

### 2.3 Measuring epistemic emotion, critical thinking, and cultural identity in social and cultural interaction

Epistemic emotions depend on dynamic learning situations. According to Pekrun et al. (2017), epistemic emotions can be identified not just in the academic setting of reading materials. Besides, how emotions affect task performance involves complex relationships with cognitive processes. Pekrun (2024) further indicates that the dimension of emotions (positive or negative) is not the only element influencing the learning outcome. The impact of emotions on task performance is achieved through the interaction of multiple mechanisms, such as “motivation, working memory, or modes of thinking.” Furthermore, emotions are associated with specific objects or situations in the external world rather than just internal physiological responses. As Whissell (2023) states, emotions are learned through various life experiences. According to Van Hemert et al. (2007), some cultures may encourage the expression of positive emotions, while others are more restrained in emotional expression. Some studies mainly explore the interwined relationships between emotions, behavior, and culture from the intercultural perspective (Evans et al., 2017; Lu et al., 2017; Mesquita et al., 2017; Gip et al., 2022; Dickter et al., 2025; Alhwaiti, 2024), or focus on emotional behavior measurement (Brown et al., 2025; Manzi et al., 2025; Quansah et al., 2024; You, 2025), cultural adaptability (Chan et al., 2024; Ebrahimabadi et al., 2024; Yang and Liu, 2025), and social problems (Ghorbanzadeh et al., 2023; Hogan and Barnes, 2024; Mesana et al., 2024; O’Keeffe, 2024). These findings suggest that emotional expressions and reactions are distinguished depending on the dynamic social and cultural contexts. Individuals’ epistemic emotions can be various in the same learning situations when dealing with cultural differences. Thus, measuring epistemic emotions requires a multifaceted approach considering cognitive ability and cultural context.

The measurement of epistemic emotions cannot be completely “emotional.” The self-report method used by Pekrun et al. (2017) lists the types of emotions involved in epistemic activities. Nevertheless, given the complexities of individuals’ rational and emotional responses to different cultures, this method may not be suitable for measuring epistemic emotions in specific cultural situations. Emotions change with situational impressions and identity expectations. According to Heise (1987), “An emotion qualifies an identity in a way that describes where the transient impression of a person is relative to the fundamental sentiment for the person’s identity.” Robinson et al. (2006) state that emotional “labels” that are culturally assigned indicate self-identity according to specific situations. These “labels” can be specified through three dimensions in social interactions: evaluation (good or bad), potency (powerful or weak), and activity (lively or weak). Moreover, according to Lively and Heise (2014), “An identity’s characteristic emotion can be viewed as the target emotion being sought by individuals enacting that identity,” indicating that emotions drive the construction and development of identity. In line with that, critical thinking is an “epistemically responsible procedure” for constructing critical identity (Marabini, 2022) in overcoming biases (Morton and Parsons, 2018), fostering critical cultural self-awareness (Cameron, 2023; Flake and Lubin, 2024), and helping individuals reflect on beliefs and behaviors in cultural adaptation (Ilyas, 2018; Kassis-Henderson et al., 2018; Morgan and Cieminski, 2023). These studies underscore the importance of critical thinking as a vital epistemic activity in constructing cultural identity. Consequently, integrating critical thinking and cultural identity should be considered when assessing epistemic emotions.

Furthermore, epistemic emotions are rarely included in critical thinking and cultural identity inventories. Measurements of critical thinking are various, such as the Watson-Glaser Critical Thinking Appraisal (WGCTA, Watson and Glaser, 1985) and the California Critical Thinking Disposition Inventory (CCTDI, Facione, 1989); other new inventories assess the attitude and belief about critical thinking, like the Critical Thinking Toolkit (CriTT, Stupple et al., 2017), the Questionnaire of Attitudes Towards Critical Thinking (QATCT, Manassero-Mas et al., 2022), and the Student-Educator Negotiated CT Dispositions Scale (SENCTDS, Quinn et al., 2020). Additionally, the inventories of identity and cultural identity are also abundant, such as Bicultural Identity Integration Scale (BIIS-1, Haritatos and Benet-Martínez, 2002), Multigroup Ethnic Identity Measure (MEIM, Phinney, 1992; Phinney and Ong, 2007), Ethnic Identity Scale (EIS, Umaña-Taylor et al., 2004), Self-Concept and Identity Measure (SCIM, Bogaerts et al., 2018), and Multigroup Ethnic & National Identity Measure (MENI, Maehler et al., 2019; Maehler et al., 2025). However, these measurements seldom contain epistemic emotions. Critical thinking application and cultural identity construction are significant in the learning process of coping with cultural differences. Therefore, in this research, the EpiCT-CI Scale is developed to offer a more comprehensive framework for exploring the complex relationships between emotions, cognition, and cultural contexts. By integrating critical thinking and cultural identity within the measurement of epistemic emotions, this scale explores how individuals’ evaluation of cultural information influences their epistemic emotions, highlighting the significant role of epistemic emotions in shaping the cognitive processes and developing cultural identity.

## 3 Materials and methods

### 3.1 Research aims

The research has three purposes. The first is to explore the related epistemic emotion in the process of comprehension, application, and reflection about critical thinking during COVID-19 in Study 1. The second is to investigate the epistemic emotions experienced by participants who conducted the task of reading and writing English articles in Study 2. The third purpose is to develop and validate the EpiCT-CI Scale. Combining the qualitative and quantitative methods, this research focuses on four research questions:

1. What kind of epistemic emotions are experienced by students during the comprehension, application, and reflection on critical thinking during the COVID-19 pandemic?
2. What epistemic emotions are experienced by EFL learners while reading and writing English articles?
3. How can the EpiCT-CI Scale be formed and developed based on the findings of Studies 1 and 2?
4. How can the EpiCT-CI Scale be validated?

### 3.2 Research design

This research employs a sequential QUAL→QUAN approach (Churchill, 1979; Creswell and Clark, 2017) to develop and validate the EpiCT-CI Scale. It includes three studies and utilizes both qualitative and quantitative analyses involving different groups of participants. Study 1 encouraged students to express perspectives and real-life experiences about how critical thinking had influenced their beliefs and actions when dealing with cultural issues exacerbated by COVID-19. Thirty participants’ essays were selected randomly for qualitative analysis on epistemic emotion. In Study 2, students were required to read two English articles before writing the essay. The narrations about epistemic emotion during the reading and writing task were submitted with the essay. Thirty-five participants’ narrations and essays were randomly selected for qualitative analysis. The item pool of the EpiCT-CI Scale was generated based on the results of Studies 1 and 2. In Study 3, the EpiCT-CI Scale was modified and validated.

### 3.3 Samples and data collection

Approximately 400 students from arts subjects and 600 from various disciplines in China were involved in this research study. Informed consent was obtained from participants before the research. In Study 1, qualitative data were collected from the writing assignments of 30 participants who were randomly selected among 300 students. In Study 2, students were required to submit narrations about epistemic emotions and writing assignments for the English class. The data from 35 participants were randomly selected to be analyzed in NVivo 15.0. Study 3 distributed the newly developed EpiCT-CI Scale among 800 students for validation.

### 3.4 Research tools

The research employs a variety of tools. In Studies 1 and 2, NVivo 15.0 analyzes the data from the selected writing assignment for college English courses. The qualitative data in Study 1 were taken from 30 essays about comprehension and reflection on the critical thinking application in COVID-19. In Study 2, two articles from the intercultural expert Roger Baumgarte were chosen as the reading materials (Baumgarte, 2016). After reading and writing, 35 randomly selected essays were analyzed. In Study 3, the EpiCT-CI Scale was refined and validated using SPSS 20.0 and AMOS 29.0.

### 3.5 The procedure of EpiCT-CI scale development and validation

The EpiCT-CI Scale is developed and validated through Studies 1, 2, and 3. The first phase of studies 1 and 2 involved identifying emotions by automatically coding. This initial step was crucial for understanding the map of individuals’ epistemic emotions on a macro level. The second phase was open coding, where the data was reviewed to generate keywords and phrases that emerged from the data. The third phase was axial coding, which categorized the child nodes regarding critical thinking application and cultural identity constructions. The final phase was selective coding, where the categories were continually integrated to form the core themes. The initial item pool was established based on child nodes and then classified according to the themes. In Study 3, after content validation, the scale was modified and validated through exploratory factor analysis (EFA) in SPSS 20.0 and confirmatory factor analysis (CFA) in AMOS 29.0. The scale’s criterion-related validity was examined through the Need for Cognition Scale (NFC) (18 items, Cacioppo et al., 1984) and the dimension of Openness in the Big Five Inventory (10 items, John et al., 2008).

## 4 Results

### 4.1 The development of the EpiCT-CI scale

#### 4.1.1 The qualitative data analysis of epistemic emotions in critical thinking applications

**Figure 1:**
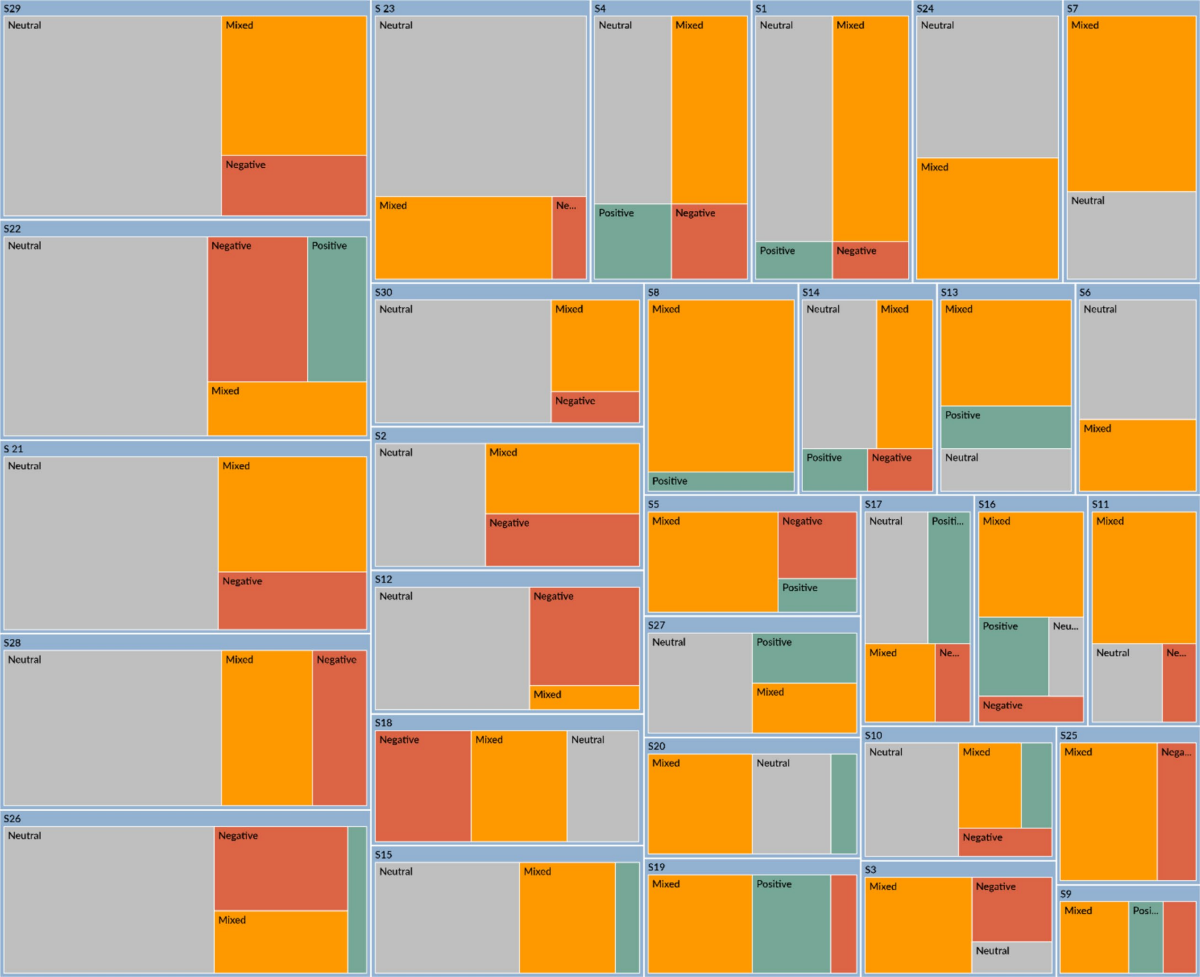
The mixed emotions of participants about critical thinking applications.

**Figure 2:**
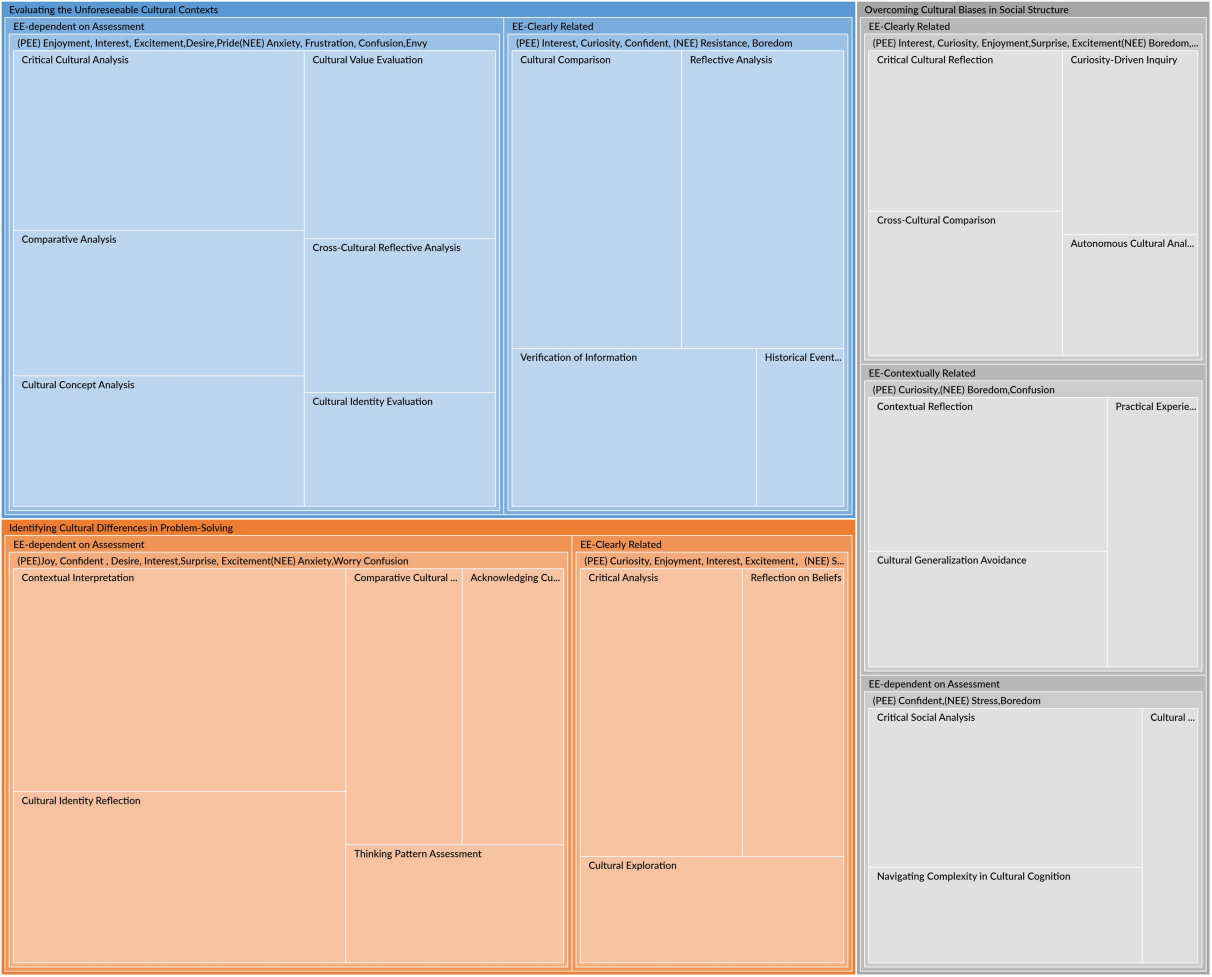
The epistemic emotion for critical thinking application.

Figures 1, 2 illustrate the epistemic emotions experienced by participants as they analyze and reflect on their experiences of applying critical thinking during COVID-19 (S1, S2,…, S30 represent the participants). As Figure 1 shows, the participants’ emotions identified automatically by NVivo 15.0 encompass neutral, mixed, positive, and negative emotions. Figure 2 demonstrates that each category of epistemic emotion-related activities is labeled as positive epistemic emotions (PEE) and negative epistemic emotions (NEE). Besides, according to Mendonça (2024), emotions have the nature of dynamic, multi-layered structures. Derived from the analysis of participants’ descriptions, related activities in applying critical thinking are also generated into different levels: EE-Clearly Related, EE-Contextually Related, and EE-Dependent on Assessment. EE-Clearly Related represents the activities that directly involve epistemic emotions in knowledge acquisition or evaluation; EE-Contextually Related represents the activities that are related to epistemic emotions only in specific contexts; EE-dependent on Assessment represents those activities that involve epistemic emotions depending on the assessment for the external or internal events. Furthermore, Quinn et al.’s (2020) SENCTDS clarifies the critical thinking dispositions into “reflection, attentiveness, open-mindedness, organization, perseverance, and intrinsic goal motivation.” These can be integrated into the epistemic activities of evaluating evidence and information, addressing cultural issues, and navigating challenging contexts. Therefore, the roles of critical thinking in cultural identity construction are categorized into three themes: “Identifying Cultural Differences in Problem-Solving,” “Evaluating the Unforeseeable Cultural Context,” and “Overcoming Cultural Biases in Social Structures.” Based on the involved epistemic emotions in each theme, 30 items of the EpiCT-CI Scale are established.

##### 4.1.1.1 Identifying cultural differences in problem-solving

“Identifying Cultural Differences in Problem-Solving (ICDPS)” is vital for applying critical thinking to cultural challenges. Figure 2 and Table 1 illustrate that this theme includes EE-Clearly Related and EE-Dependent on Assessment.

**Table 1:** The theme of identifying cultural differences in problem-solving.

Parent nodes	Child nodes	Number of nodes	Epistemic emotions	The EpiCT-CI items
EE-clearly related	Cultural Exploration	27	Curiosity	ICDPS1
	Critical Analysis	45	Stress & Boredom	ICDPS2
	Reflection on Beliefs	28	Interest & Excitement	ICDPS3
EE-dependent on assessment	Acknowledging Cultural Impacts	26	Joy & Confident	ICDPS4
	Contextual Interpretation	69	Desire & Interest	ICDPS5
	Comparative Cultural Analysis	30	Confusion & Anxiety	ICDPS6
	Thinking Pattern Assessment	24	Surprise & Excited	ICDPS7
	Cultural Identity Reflection	53	Worry & Confusion	ICDPS8

The EE-Clearly Related activities are characterized by Critical Analysis (45), Cultural Exploration (27), and Reflection on Beliefs (28). These activities represent critical thinking applications, such as analyzing, recognizing, reflecting, and comprehending, which are associated with positive epistemic emotions (curiosity, enjoyment, interest, excitement) and negative epistemic emotions (stress, boredom). Items ICDPS1, ICDPS2, and ICDPS3 are based on these results. The EE-dependent on Assessment activities involve Acknowledging Cultural Impacts (26), Comparative Cultural Analysis (30), Contextual Interpretation (69), Cultural Identity Reflection (53), and Thinking Pattern Assessment (24). These activities represent the assessing, comparing, and verifying in critical thinking applications, which are associated with a mix of positive epistemic emotions (joy, confidence, desire, interest, surprise, excitement) and negative epistemic emotions (worry, confusion, anxiety). These epistemic emotions highlight the complexity of engaging with cultural differences, where individuals may experience enthusiasm or frustration in cultural identity reflection and in navigating the dynamic and multifaceted nature of cultural differences. Based on the findings, items ICDPS4, ICDPS5, ICDPS6, ICDPS7, and ICDPS8 are created.

Based on the theme ICDPS, eight EpiCT-CI scale items can be established as follows:

- ICDPS1. I’m curious about different cultures and eager to discover why they differ.
- ICDPS2. I think using critical thinking to analyze cultural differences is stressful.
- ICDPS3. When I reflect on the differences in cultural values, I am always excited about the new insights.
- ICDPS4. Using critical thinking makes me feel more confident in different cultural situations.
- ICDPS5. I am interested in analyzing why cultural differences happen.
- ICDPS6. I am confused when I critically analyze cultural differences.
- ICDPS7. I am surprised and excited about discovering new ways of thinking in Chinese culture.
- ICDPS8. I’m worried that I am confused about my reflection of Chinese culture.

##### 4.1.1.2 Evaluating the unforeseeable cultural contexts

The theme “Evaluating the Unforeseeable Cultural Contexts (EUCC)” provides a structured approach to applying critical thinking in evaluating and analyzing cultural contexts. As Figure 2 and Table 2 show, this theme also encompasses two parent nodes related to epistemic emotion activities: EE-Clearly Related and EE-dependent on Assessment.

**Table 2:** The theme of evaluating the unforeseeable cultural contexts.

Parent nodes	Child nodes	Number of nodes	Epistemic emotions	The EpiCT-CI items
EE-clearly related	Verification of Information	36	Confident	EUCC1
	Cultural Comparison	47	Interest &Curiosity	EUCC2
	Reflective Analysis	45	Fear	EUCC3
	Historical Events Interpretation	13	Boredom	EUCC4
EE-dependent on assessment	Cultural Value Evaluation	33	Enjoyment	EUCC5
	Comparative Analysis	39	Anxiety	EUCC6
	Cultural Concept Analysis	35	Interest & Excitement	EUCC7
	Critical Cultural Analysis	48	Frustration	EUCC8
	Cultural Identity Evaluation	20	Envy	EUCC9
			Pride	EUCC10
	Cross-Cultural Reflective Analysis	27	Desire	EUCC11

Table 2 shows that four activities are in EE-Clearly Related categories: Cultural Comparison (47), Historical Events Interpretation (13), Verification of Information (36), and Reflective Analysis (45), which represent the comprehensive approach for applying critical thinking in dealing with uncertain cultural challenges. These activities involve positive epistemic emotions (interest, curiosity, confidence), which drive individuals to engage deeply with information, concepts, and historical events in different cultural contexts. Conversely, negative epistemic emotions (fear, boredom) may hinder this process. The high number of nodes (47 for Cultural Comparison and 45 for Reflective Analysis) indicates the significance of these activities in the overall framework. Items EUCC1, EUCC2, EUCC3, and EUCC4 are based on these results.

The “EE-dependent on Assessment” category contains six activities that apply critical thinking in assessing and interpreting cultural contexts: Comparative Analysis (39), Critical Cultural Analysis (48), Cultural Value Evaluation (33), Cultural Concept Analysis (35), Cultural Identity Evaluation (20), and Cross-Cultural Reflective Analysis (27). The associated positive epistemic emotions (enjoyment, interest, excitement, desire, pride) reflect the intellectual enthusiasm that arises from applying critical thinking in cross-cultural evaluations, indicating an active motivation for reflective cultural analysis. In comparison, the negative epistemic emotions (anxiety, frustration, confusion, envy) indicate potential emotional challenges and resistance in the assessment process. The high number of nodes (39 for Comparative Analysis and 48 for Critical Cultural Analysis) shows the significance of these two activities in ensuring reliable cultural analysis. EUCC5, EUCC6, EUCC7, EUCC8, EUCC9, EUCC10, and EUCC11are established in this category.

Based on the theme EUCC, 11 EpiCT-CI scale items can be created as follows:

- EUCC1. I am confident as I critically evaluate the accuracy of cultural information.
- EUCC2. I am curious and interested in critically comparing the differences between cultures.
- EUCC3. I resist critically reflecting on cultural phenomena.
- EUCC4. I find critically interpreting historical events dull and uninteresting.
- EUCC5. I enjoy critically analyzing complex issues in different cultural values.
- EUCC6. Applying critical thinking in cultural comparison makes me anxious because it is challenging.
- EUCC7. I feel interested and excited when I use critical thinking to analyze the cultural concepts.
- EUCC8. I am frustrated because using critical thinking in cultural issues analysis is challenging.
- EUCC9. I feel envious when I critically reflect on the amazing aspects of other cultures.
- EUCC10. I feel proud to critically recognize the strengths of the impressive aspects of Chinese culture.
- EUCC11. I like to evaluate the value of different cultures critically.

##### 4.1.1.3 Overcoming cultural biases in social structures

The theme “Overcoming Cultural Biases in Social Structures (OCBSS)” provides a comprehensive perspective for overcoming cultural biases. Figure 2 and Table 3 illustrate that three categories are included: EE-Clearly Related, EE-Contextually Related, and EE-dependent on Assessment.

**Table 3:** The theme of overcoming cultural biases in social structures.

Parent nodes	Child nodes	Number of nodes	Epistemic emotions	The EpiCT-CI items
EE-clearly related	Cross-Cultural Comparison	27	Surprise & Excitement	OCBSS1
	Autonomous Cultural Analysis	16	Boredom	OCBSS5
	Critical Cultural Reflection	30	Interest	OCBSS6
	Curiosity-Driven Inquiry	24	Enjoyment	OCBSS4
			Curiosity	OCBSS7
EE-contextually related	Cultural Generalization Avoidance	27	Confusion	OCBSS2
	Contextual Reflection	36	Boredom	OCBSS3
	Practical Experience Analysis	24	Curiosity	OCBSS8
EE-dependent on assessment	Cultural Identity Recognition	14	Boredom	OCBSS9
	Critical Social Analysis	43	Confident	OCBSS10
	Navigating Complexity in Cultural Cognition	26	Stress	OCBSS11

As Table 3 demonstrates, the “EE-Clearly Related” involves the activities that are directly associated with overcoming cultural biases: Cross-Cultural Comparison (27), Critical Cultural Reflection (30), Autonomous Cultural Analysis (16), and Curiosity-Driven Inquiry (24), which represent the establishment of comprehensive insights into cultural biases through critical thinking. The related positive epistemic emotions (surprise, excitement, interest, curiosity, enjoyment) drive individuals to reflect on cultural issues and evaluate cultural bias critically. In contrast, negative epistemic emotions (boredom) obstruct the process of personal understanding of cultural biases. Table 3 shows that OCBSS1, OCBSS5, OCBSS6, OCBSS4, and OCBSS7 are created in this category.

The “EE-Contextually Related” includes three activities that are significant in overcoming cultural biases: Cultural Generalization Avoidance (27), Contextual Reflection (36), and Practical Experience Analysis (24). The associated positive epistemic emotion (curiosity) encourages individuals to recognize and analyze their cultural identities and practical experiences. In contrast, the negative epistemic emotions (confusion, boredom) represent the resistance to critical analysis of cultural identity commitment. Based on this result, OCBSS2, OCBSS3, and OCBSS8 are created.

The “EE-dependent on Assessment” also includes three activities that manage the negotiations of cultural identity: Cultural Identity Recognition (14), Critical Social Analysis (43), and Navigating Complexity in Cultural Cognition (26). The related positive epistemic emotions (confidence) suggest the emotion that enhances cultural identity comprehension through critical analysis, while the related negative epistemic emotions (stress, boredom) represent unpleasant experiences when overcoming the difficulties in understanding cultural influences on identity. OCBSS9, OCBSS10, and OCBSS11 are established in this category.

Based on the theme OCBSS, 11 items of the EpiCT-CI scale can be created as follows:

- OCBSS1. I feel surprised and excited when analyzing the unexpected similarities between Chinese and Western cultures.
- OCBSS2. I am confused when critically reflecting on how to avoid misunderstandings of other cultures.
- OCBSS3. It is boring to critically examine the cultural bias in our context.
- OCBSS4. I enjoy critically examining cultural phenomena to understand them truly.
- OCBSS5. It feels boring and unnecessary to critically evaluate information from different cultures.
- OCBSS6. It is fascinating to analyze and explore the causes of cultural differences.
- OCBSS7. I am curious to explore different cultural information and verify it myself.
- OCBSS8. I am curious about recognizing and exploring the interesting aspects of Chinese culture.
- OCBSS9. It feels boring to reflect on how culture shapes my behavior.
- OCBSS10. I believe critical thinking fosters understanding of diverse cultures and encourages social harmony.
- OCBSS11. It is stressful to apply critical thinking when analyzing the complexity of Chinese culture.

#### 4.1.2 The qualitative analysis of epistemic emotions in cultural identity constructions

**Figure 3:**
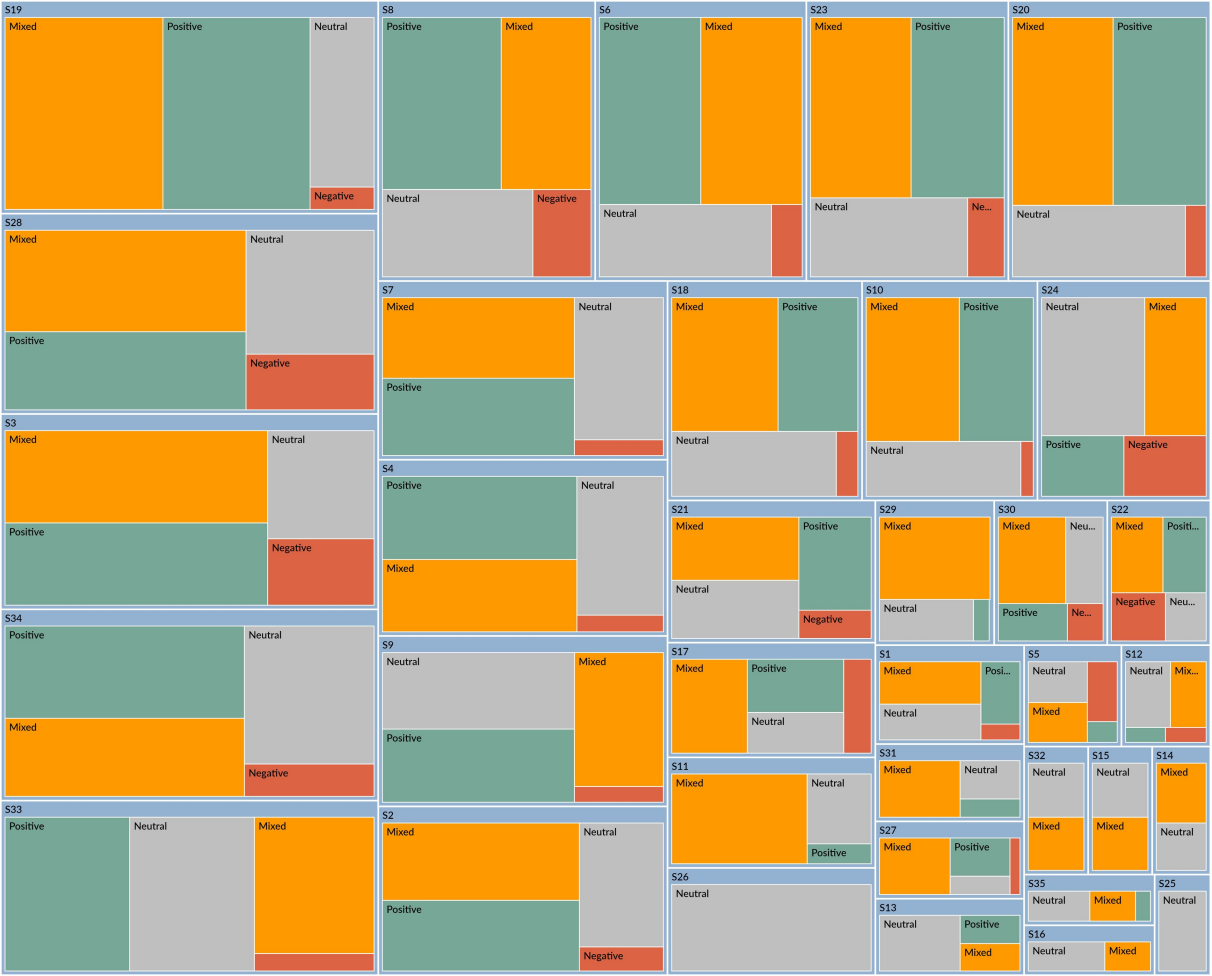
The mixed emotions of participants about cultural identity constructions.

**Figure 4:**
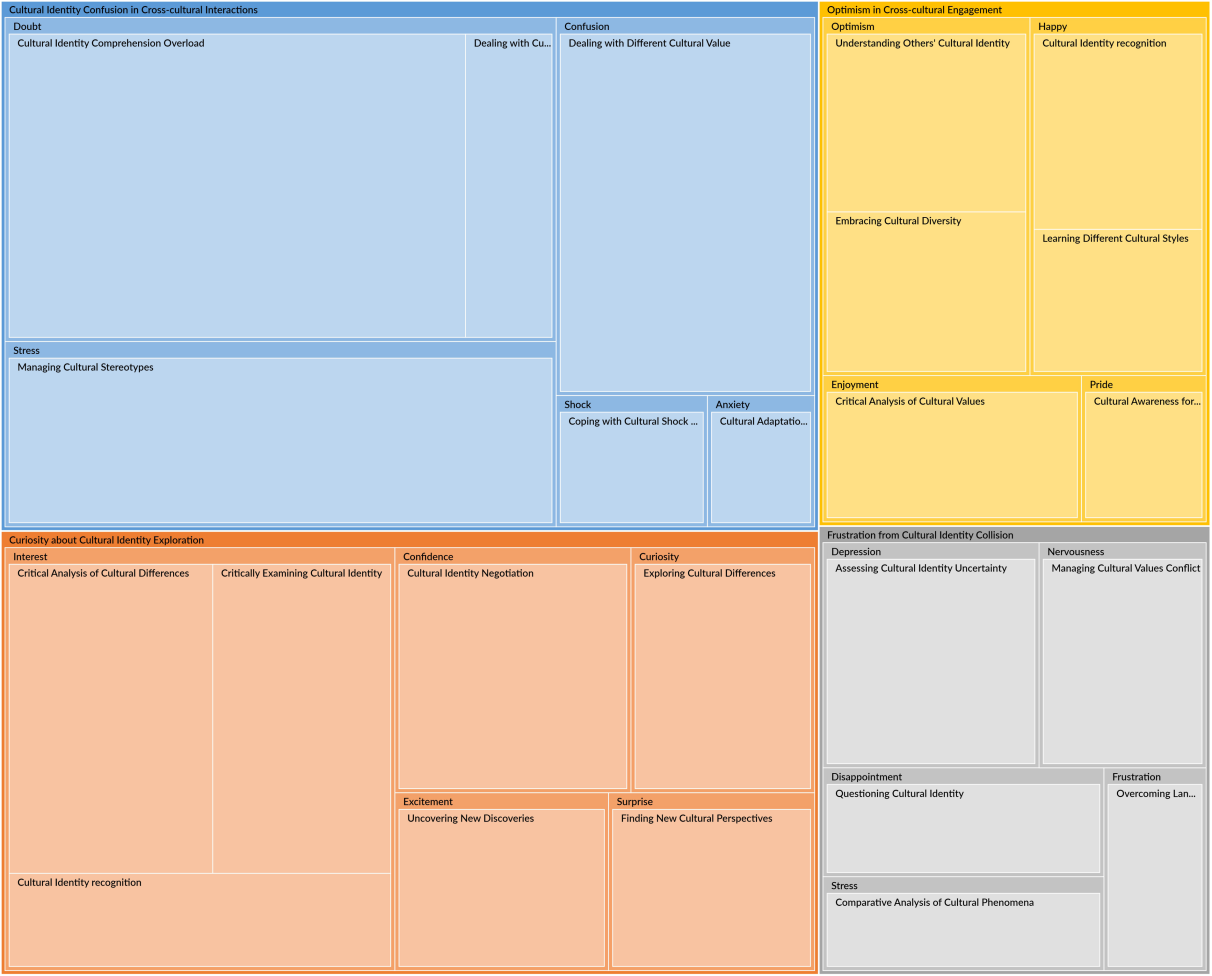
The epistemic emotion for cultural identity constructions.

The result of Study 2 is shown in Table 4 and Figures 3, 4. Study 2 was conducted differently from Study 1. In a regular writing class of a college English course, participants are assigned to describe their opinions and feelings about friendship. Friendship is an interesting and relatable topic that represents the internal and external relationships between individuals and the world around them. The students are provided with two articles by Roger Baumgarte, which discuss how cultural differences influence friendships. One is a reading assignment in our English course textbook, which is excerpted from Roger’s book, Friends Beyond Borders. The other is a chapter titled “Interveners and Independents” from his research paper (Baumgarte, 2016). These two articles are at an average level of English, and participants are encouraged to express their specific emotions through reading and writing. Figure 3 is the sentiment coding from NVivo 15.0 for the emotions of 35 participants (S1, S2, S3…, S35). Compared with Figure 1, Figure 3 shows fewer negative emotions; most participants express positive, mixed, or neutral emotions. This result suggests that when participants relate cultural differences to real life, they think positively and neutrally.

**Table 4:** Epistemic emotions related to cross-cultural understanding.

Themes	Parent nodes	Child nodes	Number of nodes	The EpiCT-CI items
Cultural identity confusion in cross-cultural interactions	Anxiety	Cultural Adaptation Challenges	10	CICCI1
	Confusion	Dealing with Different Cultural Values	69	CICCI2 (ICDPS6)
	Doubt	Cultural Identity Comprehension Overload	106	CICCI3
		Dealing with Cultural Beliefs	20	CICCI4
	Stress	Managing Cultural Stereotypes	72	CICCI5
	Shock	Coping with Cultural Shock and Identity	14	CICCI6
Curiosity about cultural identity exploration	Interest	Cultural Identity recognition	27	CCIE1
		Critically Examining Cultural Identity	42	CCIE3
		Critical Analysis of Cultural Differences	48	CCIE6
	Curiosity	Exploring Cultural Differences	32	CCIE2
	Surprise	Finding New Cultural Perspectives	26	CCIE4
	Confidence	Cultural Identity Negotiation	41	CCIE5
	Excitement	Uncovering New Discoveries	27	CCIE7
Frustration from cultural identity collision	Depression	Assessing Cultural Identity Uncertainty	35	FCIC1
	Stress	Comparative Analysis of Cultural Phenomena	19	FCIC2
	Disappointment	Questioning Cultural Identity	22	FCIC3
	Nervousness	Managing Cultural Values Conflict	27	FCIC4
	Frustration	Overcoming Language Barriers	15	FCIC5
Optimism in cross-cultural engagement	Optimism	Embracing Cultural Diversity	25	OCCE1
		Understanding Others’ Cultural Identity	28	OCCE3
	Pride	Cultural Awareness for Personal Growth	13	OOCE2
	Happy	Cultural Identity Recognition	26	OCCE4
		Learning Different Cultural Styles	19	OCCE5
	Enjoyment	Critical Analysis of Cultural Values	27	OCCE6(EUCC5)

Table 4 and Figure 4 illustrate the four main themes of epistemic emotion groups when dealing with cultural identity challenges: Cultural Identity Confusion in Cross-cultural Interactions, Curiosity about Cultural Identity Exploration, Frustration from Cultural Identity Collision, and Optimism in Cross-cultural Engagement. Each theme encompasses various epistemic emotions and activities, including thinking, analyzing, and evaluating cultural impacts. According to the research findings, 22 items are newly created based on every activity associated with each epistemic emotion, with the other two shared items from Study 1 (ICDPS6, EUCC5).

##### 4.1.2.1 Cultural identity confusion in cross-cultural interactions

Table 4 and Figure 4 show that the theme of Cultural Identity Confusion in Cross-cultural Interactions (CICCI) encompasses four types of negative epistemic emotions: Anxiety, Confusion, Doubt, Stress, and Shock. Cross-cultural interactions would cause these mixed feelings of confusion.

*S6: The article’s labeling of the intervenor and the independent as representations of Eastern and Western friendship confuses me because I have realized that friendship cannot always be defined rigidly as interventionist or independent. I also feel stressed, for it is anxious to imagine myself in a friendship with someone from a completely different culture.*

*S13: The article’s exploration of the boundaries of friendship across different cultures has sometimes confused me, such as in the interventionist style. Understanding complex cultural differences in friendships causes stress and may lead to inaccuracies in my writing and practical applications.*

The parent node Anxiety includes Cultural Adaptation Challenges (10). Confusion involves Dealing with Different Cultural values (69). Doubt involves Dealing with Cultural Beliefs (20) and Cultural Identity Comprehension Overload (106). Stress involves Managing Cultural Stereotypes (69). Shock includes Coping with Cultural Shock and Identity (14). These epistemic emotions demonstrate the mixed feelings that participants experience in interpersonal interactions in different cultures when constructing cultural identity. Based on every activity associated with each epistemic emotion, items CICCI1, CICCI2, CICCI3, CICCI4, CICCI5, and CICCI6 of the EpiCT-CI Scale are created. Due to the epistemic emotion “Confusion” (Dealing with Different Cultural Values) in this theme being closely connected to “Confusion & Anxiety” (Comparative Cultural Analysis) in the theme ICDPS of Study 1, these two nodes are linked by the shared item CICCI2 (ICDPS6).

- CICCI1. I feel anxious when I struggle with cultural adaptation challenges.
- CICCI2. I am confused when trying to understand different cultural values.
- CICCI3. I doubt my ability to truly understand unfamiliar cultural traditions when overwhelmed by their complexity.
- CICCI4. It is confusing to deal with a variety of cultural beliefs.
- CICCI5. I feel stressed when dealing with stereotypes about a certain group.
- CICCI6. I was in shock at how different everything is from the culture I grew up in.

##### 4.1.2.2 Curiosity about cultural identity exploration

As illustrated in Table 4 and Figure 4, the Curiosity about Cultural Identity Exploration (CCIE) theme comprises five positive epistemic emotions: Interest, Curiosity, Confidence, Excitement, and Surprise. The parent node Interest includes Cultural Identity recognition (27), Critically Examining Cultural Identity (42), and Critical Analysis of Cultural Differences (48). Curiosity includes Exploring Cultural Differences (32). Confidence includes Cultural Identity Negotiation (41). Excitement includes Uncovering New Discoveries (27). Surprise includes Finding New Cultural Perspectives (26). This theme primarily concerns the participants’ positive epistemic emotions when discovering, exploring, and evaluating new cultural knowledge and perspectives in constructing cultural identities.

*S19: I have developed a strong interest in different cultural values. I am also excited by the various behavioral styles arising from cultural differences. Exploring these differences sparks my interest and desire to think.*

*S20: When reading this article, I became intensely interested in how cultural identity influences friendships. I was also surprised to learn for the first time about the concepts of ‘interferers’ and ‘independents.’ The idea of an “interferer” has challenged my conventional understanding of what constitutes a true friendship.*

Items CCIE1, CCIE2, CCIE3, CCIE4, CCIE5, CCIE6, and CCIE7 are established in this theme.

- CCIE1. Understanding my cultural traditions is interesting and helps me understand who I am.
- CCIE2. Curiosity often drives me to explore the differences between various cultures.
- CCIE3. It is interesting to comprehend those unique values from different cultural backgrounds.
- CCIE4. It is amazing to discover new cultural phenomena because they give me new ways of thinking.
- CCIE5. I am confident in my ability to handle conflicts between different cultural identities.
- CCIE6. I am interested in critically examining the history and traditions of different cultures.
- CCIE7. It is exciting to find something new when encountering new cultural phenomena.

##### 4.1.2.3 Frustration from cultural identity collision

Table 4 and Figure 4 show that the theme of Frustration from Cultural Identity Collision (FCIC) comprises five negative epistemic emotions: Disappointment, Depression, Frustration, Nervousness, and Stress, which reflect various kinds of annoyance when dealing with cultural challenges.

*S11: I am disappointed that the article simplistically considers Western friendships as ‘independent’ style and Eastern friendships as ‘interfering’. I’ve seen many Western friends actively helping others in tough times, while some Easterners prefer independence. I also felt frustrated about conveying my message in English because my thoughts were lost in that language.*

*S34: I feel stressed by friendships involving cultural differences. I have experienced a typical case. A classmate has lived abroad since elementary school, and I’m nervous about our interaction for fear of offending due to these differences.*

Disappointment includes Questioning Cultural Identity (22). Frustration includes Overcoming Language Barriers (15). Nervousness includes Managing Cultural Values Conflict (27). Stress includes Comparative Analysis of Cultural Phenomena (19). Depression includes Assessing Cultural Identity Uncertainty (35). According to Pekrun et al. (2017), frustration is a negative epistemic emotion like confusion. However, the findings of Study 2 reveal that these two emotions contain different epistemic activators in cross-cultural challenges. Items FCIC1, FCIC2, FCIC3, FCIC4, and FCIC5 are established in this theme.

- FCIC1. The prejudices some people have about Chinese culture depress me.
- FCIC2. I feel more relaxed when I interact with people who share the same cultural background as I do.
- FCIC3. I feel disappointed because some aspects of other cultures are not what I expected.
- FCIC4. I feel nervous when attempting to understand how people from different cultural backgrounds think.
- FCIC5. I feel frustrated when communicating in English because expressing myself is hard.

##### 4.1.2.4 Optimism in cross cultural engagement

Table 4 and Figure 4 illustrate that Optimism in Cross-cultural Engagement (OCCE) represents the pleasure of dealing with cultural challenges, which comprises Enjoyment, Happy, Optimism, and Pride. Enjoyment includes Critical Analysis of Cultural Values (25). Happy includes Cultural Identity Recognition (26) and Learning Different Cultural Styles (19). Optimism includes Understanding Others’ Cultural Identity (28) and Embracing Cultural Diversity (25). Pride includes Cultural Awareness for Personal Growth (13). These epistemic emotions represent the participants’ delightful feeling, which reflects a sense of accomplishment and a deeper appreciation of their cultural identity. These positive experiences stem from overcoming cultural barriers in constructing cultural identity.

*S8: I enjoy comparing how different cultures define friendship. My optimism about the feasibility of cross-cultural friendship directly influenced the ending of my writing. Cultural differences are not a barrier. I encourage everyone to accept the pluralistic view of friendship with an open mind.*

*S11. I am proud of my Eastern-style friendship, which is rich in kindness and collective strength. It demonstrates unique value and charm, making me proud of my culture. I am also optimistic that cultural differences are not the root of conflict, which fills me with confidence about cross-cultural friendships.*

Items OCCE1, OCCE3, OCCE2, OCCE4, OCCE5, and OCCE6 are created based on the result. Because the epistemic emotion “Enjoyment” (Critical Analysis of Cultural Values) in this theme is aligned with “Enjoyment” (Cultural Value Evaluation) in the theme EUCC of Study 1, these two nodes are linked by the shared item OCCE6 (EUCC5).

- OCCE1. I appreciate the different values and lifestyles that arise from cultural diversity.
- OCCE2. I am proud to appreciate the values and traditions of different cultures, as they help me grow.
- OCCE3. I view cultural diversity optimistically because the differences between cultures enrich our perspectives.
- OCCE4. After critically rethinking traditional beliefs, I am happy to gain new insights into culture and tradition.
- OCCE5. I am happy to learn about different cultural lifestyles in this wonderful and interesting world.
- OCCE6. I enjoy critically analyzing challenges that arise from different cultural values.

### 4.2 The validation of EpiCT-CI scale

#### 4.2.1 Content validity

The initial 52 EpiCT-CI Scale item pool consists of 30 items from Study 1 and 22 from Study 2. According to Mayer (2025), critical thinking skills and identity develop through cross-cultural conflicts, as individuals reflect on different perspectives and potential actions. Tables 1–3 in the findings of Study 1, 30 critical thinking items in the EpiCT-CI Scale are generated into three parts: ICDPS (8 items), EUCC (11 items), and OCBSS (11 items). Table 4 in the findings of Study 2 shows 24 items of cultural identity constructions that are classified into four parts: CICCI (5 items), CCIE (7 items), FCIC (5 items), and OCCE (5 items). Seven experts who had not participated in data collection or authorship evaluated the items in the content validation process. Four items (EUCC11, OCBSS9, CICCI4, CICCI5) are deleted after assessment, and 48 items are left for scale modification.

#### 4.2.2 Scale modification

The EFA was conducted to refine the items and adjust the dimensional structure of the EpiCT-CI scale. After content validation, the initial EpiCT-CI Scale of 48 items is rated on a five-point Likert scale (1 = strongly disagree, 2 = disagree, 3 = neutral, 4 = agree, 5 = strongly agree). Three hundred and 10 questionnaires were distributed online to students of different subjects in an arts college. Females constituted the majority (*n* = 249, 80.3%), whereas males accounted for 19.7% (*n* = 61) of the sample. The distribution of respondents across nine undergraduate majors in the arts college is as follows: Product Design (*n* = 95, 30.6%), Fashion & Accessories Design (*n* = 13, 4.2%), Industrial Design (*n* = 18, 5.8%), Painting (*n* = 50, 16.1%), Science & Art (*n* = 37, 11.9%), Experimental Art (*n* = 21, 6.8%), Calligraphy (*n* = 9, 2.9%), Artworks Conservation and Restoration (*n* = 16, 5.2%), and Intelligent Interaction Design (*n* = 51, 16.5%).

Table 5 demonstrates that the reliability of this initial 48-item scale is excellent (Cronbach’s *α* = 0.955, standardized *α* = 0.961). Across every item, the “Cronbach’s Alpha if Item Deleted” remains within an extremely tight band, 0.954 < *α* < 0.956. The corrected item-total correlations span a considerably wider interval, with 24 items falling in the high-correlation band (0.60 ≤ *r* ≤ 0.68), 20 items in the moderate band (0.45 ≤ *r* < 0.60), and only four items in the lower band (*r* < 0.45). Thus, the scale mainly consists of items that are closely related to the total score, with no single item undermining the instrument’s high internal consistency.

**Table 5:** Descriptive statistical analysis of initial EpiCT-CI Scale.

Item	Mean	Item-total correlation	Cronbach’s *α* if item deleted	Skewness		Kurtosis	
				Statistic	Std. Error	Statistic	Std. Error
CCIE1	3.93	0.571	0.954	−0.207	0.138	−0.720	0.276
CCIE2	3.95	0.631	0.954	−0.249	0.138	−0.628	0.276
CCIE3	4.06	0.531	0.954	−0.327	0.138	−0.581	0.276
CCIE4	3.97	0.640	0.954	−0.181	0.138	−0.416	0.276
CCIE5	3.52	0.584	0.954	0.120	0.138	−0.346	0.276
CCIE6	3.88	0.657	0.954	−0.253	0.138	−0.620	0.276
CCIE7	4.00	0.676	0.954	−0.128	0.138	−0.454	0.276
CICCI6	3.86	0.589	0.954	−0.399	0.138	0.022	0.276
OCCE1	4.26	0.488	0.955	−0.648	0.138	0.171	0.276
OCCE2	4.00	0.673	0.954	−0.160	0.138	−0.581	0.276
OCCE3	4.10	0.620	0.954	−0.358	0.138	−0.290	0.276
OCCE4	4.04	0.634	0.954	−0.495	0.138	0.369	0.276
OCCE5	4.12	0.655	0.954	−0.444	0.138	−0.250	0.276
CICCI1	3.07	0.428	0.955	0.150	0.138	−0.374	0.276
CICCI3	3.41	0.419	0.955	−0.214	0.138	−0.275	0.276
FCIC1	3.84	0.437	0.955	−0.305	0.138	−0.501	0.276
FCIC2	4.01	0.593	0.954	−0.430	0.138	−0.010	0.276
FCIC3	3.53	0.504	0.954	−0.085	0.138	−0.391	0.276
FCIC4	3.62	0.585	0.954	−0.215	0.138	−0.254	0.276
FCIC5	4.01	0.559	0.954	−0.485	0.138	0.002	0.276
ICDPS1	3.88	0.679	0.954	−0.205	0.138	−0.394	0.276
ICDPS2	3.32	0.442	0.955	−0.172	0.138	−0.548	0.276
ICDPS3	3.87	0.637	0.954	−0.343	0.138	−0.016	0.276
ICDPS4	3.87	0.660	0.954	−0.120	0.138	−0.475	0.276
ICDPS5	3.89	0.677	0.954	−0.416	0.138	0.443	0.276
ICDPS6	3.94	0.527	0.954	−0.451	0.138	0.273	0.276
ICDPS7	3.96	0.683	0.954	−0.246	0.138	−0.529	0.276
ICDPS8	3.68	0.625	0.954	−0.368	0.138	−0.172	0.276
EUCC1	3.82	0.665	0.954	−0.100	0.138	−0.443	0.276
EUCC2	3.98	0.679	0.954	−0.179	0.138	−0.473	0.276
EUCC3	2.89	0.311	0.956	0.074	0.138	−1.011	0.276
EUCC4	2.92	0.282	0.956	0.068	0.138	−0.704	0.276
EUCC5	3.85	0.629	0.954	−0.255	0.138	0.181	0.276
EUCC6	2.95	0.349	0.955	0.097	0.138	−0.414	0.276
EUCC7	3.78	0.669	0.954	−0.007	0.138	−0.568	0.276
EUCC8	3.27	0.516	0.954	0.025	0.138	−0.506	0.276
EUCC9	3.67	0.526	0.954	−0.416	0.138	0.169	0.276
EUCC10	4.00	0.582	0.954	−0.239	0.138	−0.731	0.276
OCBSS1	3.90	0.684	0.954	−0.043	0.138	−0.508	0.276
OCBSS2	3.25	0.467	0.955	0.070	0.138	−0.188	0.276
OCBSS3	2.76	0.296	0.956	0.158	0.138	−0.903	0.276
OCBSS4	3.79	0.634	0.954	−0.102	0.138	−0.474	0.276
OCBSS5	2.95	0.308	0.956	0.111	0.138	−0.652	0.276
OCBSS6	3.92	0.660	0.954	−0.263	0.138	−0.369	0.276
OCBSS7	3.87	0.668	0.954	−0.242	0.138	−0.440	0.276
OCBSS8	3.97	0.595	0.954	−0.212	0.138	−0.421	0.276
OCBSS10	4.05	0.592	0.954	−0.239	0.138	−0.479	0.276
OCBSS11	3.51	0.597	0.954	−0.110	0.138	−0.441	0.276

Additionally, Table 5 shows that the mean scores for the items range from 2.89 to 4.26, indicating a generally positive response pattern. The skewness values for all items are within the range of −0.648 to 0.150, and the kurtosis values range from −1.011 to 0.443, indicating that the distribution of responses for each item is reasonably close to normal. The standard errors for skewness and kurtosis are 0.138 and 0.276, respectively, further supporting the stability of these descriptive statistics. Besides, the Harman single-factor method was applied to evaluate common method variance. An unrotated principal-axis factor explained 37.655% of the total variance, well below the 50% threshold (Kock et al., 2021). This indicates that common method bias is unlikely to threaten the validity of the findings.

After the reliability assessment, the data gathered from 310 samples were subjected to EFA. Given that the scale consists of 48 items, a sample size of 310 respondents is deemed adequate for EFA, adhering to the recommended guideline of 5–10 respondents per item (Rouquette and Falissard, 2011; Goretzko et al., 2021). As shown in Table 6, the Kaiser-Meyer-Olkin (KMO) (Kaiser, 1974) value remained at 0.946 after eight iterations of item refinement. Bartlett’s test of sphericity remained highly significant, *χ*^2^(528) = 6,481.942, *p* < 0.001. This result indicates that the questionnaire is suitable for factor analysis, confirming that the 33-item scale refinement was reasonable and robust.

**Table 6:** Exploratory factor analysis (*n* = 310).

Latent variables	Observed	Loading	Cronbach’s *α*	Variance explained
KMO = 0.946, Bartlett’s test of sphericity *χ*^2^(528) = 6,481.942, *p* < 0.001			0.937	60.39%
Factor 1 Joy in Critical Cultural Inquiry (JCCI)	JCCI1. Happy, Learning Different Cultural Styles (OCCE5)	0.570	0.948	40.75%
	JCCI2. Joy & Confidence, Acknowledging Cultural Impacts (ICDPS4)	0.588		
	JCCI3. Surprise &Excited, Thinking Pattern Assessment (ICDPS7)	0.590		
	JCCI4. Confident, Verification of Information (EUCC1)	0.634		
	JCCI5. Confident, Critical Social Analysis (OCBSS10)	0.664		
	JCCI6. Curiosity, Curiosity-Driven Inquiry (OCBSS7)	0.667		
	JCCI7. Interest & Curiosity, Cultural Comparison (EUCC2)	0.669		
	JCCI8. Surprise & Excitement, Cross-Cultural Comparison (OCBSS1)	0.692		
	JCCI9. Interest, Critical Cultural Reflection (OCBSS6)	0.728		
	JCCI10. Interest & Excitement, Reflection on Beliefs (ICDPS3)	0.771		
	JCCI11. Curiosity, Cultural Exploration (ICDPS1)	0.815		
	JCCI12. Enjoyment, Curiosity-Driven Inquiry (OCBSS4)	0.885		
	JCCI13. Desire & Interest, Contextual Interpretation (ICDPS5)	0.887		
	JCCI14. Interest & Excitement, Cultural Concept Analysis (EUCC7)	0.92		
	JCCI15. Enjoyment, Cultural Value Evaluation (EUCC5)	0.933		
Factor2 Boredom in Critical Cultural Reflection (BCCR)	BCCR1. Boredom, Autonomous Cultural Analysis (OCBSS5)	0.816	0.868	11.25%
	BCCR2. Boredom, Historical Events Interpretation (EUCC4)	0.744		
	BCCR3. Fear, Reflective Analysis (EUCC3)	0.823		
	BCCR4. Stress & Boredom, Critical Analysis (ICDPS2)	0.535		
	BCCR5. Boredom, Contextual Reflection (OCBSS3)	0.769		
Factor 3 Curiosity in Cultural Identity Reflection (CCIR)	CCIR1. Surprise, Finding New Cultural Perspectives (CCIE4)	0.512	0.892	4.24%
	CCIR2. Curiosity, Exploring Cultural Differences (CCIE2)	0.913		
	CCIR3. Interest, Cultural Identity recognition (CCIE1)	0.778		
	CCIR4. Pride, Cultural Awareness for Personal Growth (OCCE2)	0.522		
	CCIR5. Interest, Critical Analysis of Cultural Differences (CCIE6)	0.478		
	CCIR6. Interest, Critically Examining Cultural Identity (CCIE3)	0.711		
	CCIR7. Optimism, Embracing Cultural Diversity (OCCE1)	0.694		
Factor 4 Distress in Cultural Adaptation (DCA)	DCA1. Disappointment, Questioning Cultural Identity (FCIC3)	0.555	0.786	4.15%
	DCA2. Shock, Coping with Cultural Shock and Identity (CICCI6)	0.416		
	DCA3. Anxiety, Cultural Adaptation Challenges (CICCI1)	0.685		
	DCA4. Confusion & Anxiety, Comparative Cultural Analysis (ICDPS6)	0.430		
	DCA5. Worry& Confusion, Cultural Identity Reflection (ICDPS8)	0.425		
	DCA6. Nervousness, Managing Cultural Values Conflict (FCIC4)	0.851		

The EFA was conducted using Principal Axis Factoring (PAF) with PROMAX rotation to identify underlying dimensions within a set of observed variables related to epistemic emotions. Among 48 items, 15 items (EUCC6, EUCC8, EUCC9, EUCC10, OCBSS2, OCBSS8, OCBSS11, CCIE3, CCIE5, CCIE7, FCIC1, FCIC2, FCIC5, OCEE3, OCCE4) were excluded due to having factor loadings below 0.40 and displaying high cross-loadings on multiple factors, leaving 33 items for subsequent confirmatory factor analysis (CFA). According to Finch (2013), constructing 2–4 factors in EFA is appropriate. Table 6 shows that four factors were formed in this process: Joy in Critical Cultural Inquiry (JCCI, 15 items), Boredom in Critical Cultural Reflection (BCCR, five items), Curiosity in Cultural Identity Reflection (CCIR, seven items), and Distress in Cultural Adaptation (DCA, six items). The scale demonstrates excellent internal consistency, with a Cronbach’s alpha 0.937 for the overall 33 items. The total variance explained by the four factors is 60.39%, suggesting that these factors capture a substantial portion of the variability in the observed variables. All 33 items considered for the CFA demonstrated factor loadings that exceeded 0.40. Thirty items exhibited loadings between 0.50 and 0.90, indicating their substantial contribution to the respective factors. Only three items had loadings ranging from 0.416 to 0.478, indicating that these items still meet the threshold suggested for retention when the sample size exceeds 200 (Sürücü et al., 2022). The reliability of the factors was further assessed using Cronbach’s *α*, with values exceeding 0.8 for Factors 1, 2, 3, and 0.748 for Factor 4, suggesting strong internal consistency across all factors.

Table 6 also illustrates that Factors 1 and 2 (JCCI, BCCR) contain most items from Study 1 (ICDPS, EUCC, OCBSS). Factors 3 and 4 (CCIR, DCA) comprise most items from Study 2 (CCIE, BCCR, FCIC, OCCE). This result indicates that Studies 1 and 2 are complementary sources for this scale. The data collection of the two studies has successfully converged into a unified four-factor structure. This alignment enhances the convergent validity of the instrument and emphasizes the complementary roles of Studies 1 and 2, which effectively capture the comprehensive range of epistemic emotions in diverse cultural contexts.

#### 4.2.3 Confirmatory factor analysis (CFA) of the EpiCT-CI scale

The EpiCT-CI Scale of 33 items was validated through CFA. The questionnaires were distributed to another group of 486 students with different subjects, not limited to the art subjects. Two hundred thirty-one are male, accounting for 47.5%, while 255 are female, making up 52.5%. Specifically, the sample included students from both social sciences and STAM, such as law (*n* = 45 students, 9.3%), international relations (*n* = 22, 4.5%), finance (*n* = 132, 27.2%), art management (*n* = 10, 2.1%), art education (*n* = 13, 2.7%), product design (*n* = 28, 5.8%), mechanical engineering (*n* = 33, 6.8%), architecture (*n* = 17, 3.5%), educational technology (*n* = 29, 6.0%), computer science (*n* = 141, 29.0%), and visual communication design (*n* = 16, 3.3%). AMOS 29.0 was applied to test the 4-factor structure established in EFA (Table 6), running with a maximum likelihood estimator. Table 7 shows that the CFA yielded a four-factor structure with 19 items. Fourteen items (OCCE5, OCBSS10, OCBSS7, OCBSS6, OCBSS4, EUCC7, ICDPS2, CCIE4, OCCE2, CCIE6, CCIE3, CICCI1, ICDPS6, ICDPS8) were deleted in this process. Besides, the EpiCT-CI Scale is supposed to assess the epistemic emotions that individuals experience when actively applying critical thinking and exploring cultural identities. To ensure the scale maintains its intended directionality, the items in Factor 2 (BCCR) and Factor 4 (DCA), which reflect negative emotional valence, were reverse scored (DeVellis, 2017).

**Table 7:** The result of confirmatory factor analysis (*n* = 486).

Construct	Items	Factor loading	Cronbach’s *α*	AVE	C.R.
Factor 1: JCCI	JCCI1. Using critical thinking makes me feel more confident in different cultural situations. (ICDPS4)	0.748	0.909	0.560	0.915
	JCCI2. I am confident as I critically evaluate the accuracy of cultural information. (EUCC1)	0.770			
	JCCI3. When I reflect on the differences in cultural values, I am always excited about the new insights. (ICDPS3)	0.788			
	JCCI4. I’m curious about different cultures and eager to discover why they differ. (ICDPS1)	0.676			
	JCCI5. I enjoy critically analyzing complex issues in different cultural values. (EUCC5)	0.740			
	JCCI6. I am interested in analyzing why cultural differences happen. (ICDPS5)	0.763			
	JCCI7. I am curious and interested in critically comparing the differences between cultures. (EUCC2)	0.758			
	JCCI8. I am surprised and excited about discovering new ways of thinking in Chinese culture. (ICDPS7)	0.637			
	JCCI9. I feel surprised and excited when analyzing the unexpected similarities between Chinese and Western cultures. (OCBSS1)	0.648			
Factor 2: BCCR	BCCR1. It feels boring and unnecessary to critically evaluate information from different cultures. (OCBSS5)	0.671	0.832	0.564	0.834
	BCCR2. It is boring to critically examine the cultural bias in our context. (OCBSS3)	0.709			
	BCCR3. I find critically interpreting historical events dull and uninteresting. (EUCC4)	0.792			
	BCCR4. I resist critically reflecting on cultural phenomena. (EUCC3)	0.789			
Factor 3: CCIR	CCIR1. Understanding my cultural traditions is interesting and helps me understand who I am. (CCIE1)	0.758	0.813	0.605	0.818
	CCIR2. Curiosity often drives me to explore the differences between various cultures. (CCIE2)	0.798			
	CCIR3. I appreciate the different values and lifestyles that arise from cultural diversity. (OCCE1)	0.753			
Factor 4: DCA	DCA1. I feel nervous when attempting to understand how people from different cultural backgrounds think. (FCIC4)	0.803	0.787	0.559	0.790
	DCA2. I feel disappointed because some aspects of other cultures are not what I expected. (FCIC3)	0.694			
	DCA3. I was in shock at how different everything is from the culture I grew up in. (CICCI6)	0.731			

Table 7 shows that the retained items have standardized indicator loadings ranging from 0.637 to 0.803, exceeding the recommended threshold of 0.5–0.7 for strong relations with the associated constructs recommended by Hair et al. (2019). Additionally, each construct demonstrates strong internal consistency, with Cronbach’s *α* ranging from 0.787 to 0.909 and composite reliability (CR) from 0.790 to 0.915, all surpassing the 0.70 criterion. Average variance extracted (AVE) values range from 0.559 to 0.605, exceeding the 0.50 threshold (Hair et al., 2019), confirming convergent validity. Across all constructs, every retained indicator loaded decisively on its designated latent factor, with standardized coefficients ranging from *λ* = 0.637 to *λ* = 0.803, comfortably surpassing the 0.708 threshold Hair et al. (2019) recommended for high-quality measurement. At the construct level, JCCI (9 items, Cronbach’s *α* = 0.909, CR = 0.915, AVE = 0.560), BCCR (4 items, Cronbach’s *α* = 0.832, CR = 0.834, AVE = 0.564), CCIR (3 items, Cronbach’s *α* = 0.813, CR = 0.818, and AVE = 0.605) and DCA (3 items, Cronbach’s *α* = 0.787, CR = 0.790, and AVE = 0.559) confirm strong internal consistency and dimensional integrity. With unequal group sizes (Arts *n* = 67; non-arts *n* = 419), Tucker’s *φ* was additionally computed between the standardized 19-item/four-factor loadings obtained from the 310 arts students in the EFA sample and those derived from the 419 non-arts students in the CFA sample. The resulting value of 0.984 exceeds the ≥0.95 criterion for factorial congruence (Lorenzo-Seva and ten Berge, 2006), demonstrating that the basic factor structure is highly similar across the two independent, discipline-different groups despite their majors.

Table 8 shows the discriminant validity of the four-factor construction. The inter-constructed correlations among the associated factors reveal both positive (JCCI, CCIR: *r* = 0.617; BCCR, DCA: *r* = 0.473) and negative relationships (JCCI, BCCR: *r* = −0.624; JCCI, DCA: *r* = −0.581; BCCR, CCIR: *r* = −0.217; CCIR, DCA: *r* = −0.557). The square roots of the AVE for JCCI, BCCR, CCIR, and DCA range from 0.738 to 0.778, all exceeding the absolute values of the inter-construct correlations among the associated factors. This finding indicates that each dimension is distinct, confirming the discriminant validity for the CFA model.

**Table 8:** Discriminant validity: Pearson correlations and AVE square roots.

Factors	JCCI	BCCR	CCIR	DCA
JCCI	0.738			
BCCR	−0.624^***^	0.748		
CCIR	0.617^***^	−0.217^***^	0.778	
DCA	−0.581^***^	0.473^***^	−0.557^***^	0.747

*p* < 0.001.

As Table 9 demonstrated, the CFA result has a good model fit: *χ*^2^ = 385.748, df = 146, *p* < 0.001, *χ*^2^/df = 2.642, GFI = 0.917, RMSEA = 0.058, RMR = 0.066, SRMR = 0.0466, CFI = 0.947, NFI = 0.918, and NNFI = 0.938. With CFI ≥ 0.94, the standardized RMR (SRMR = 0.0466) is well below the 0.05 criterion for excellent fit (Hair et al., 2019). Besides, a one-factor model was specified as a stringent test of discriminant validity. The constrained solution yielded a *χ*^2^/df of 9.00, RMSEA = 0.128 (90% CI 0.122–0.135), SRMR = 0.099, and CFI = 0.731, all lying outside the thresholds recommended for adequate fit (*χ*^2^/df < 3, RMSEA ≤ 0.08, SRMR ≤ 0.08, CFI ≥ 0.90). The comparison between the one-factor and four-factor models shows compelling evidence for the discriminant validity of the scale.

**Table 9:** Model fit indication.

Model	*X* ^2^	df	*p*	*χ*^2^/df	GFI	RMSEA	RMR	SRMR	CFI	NFI	NNFI
	–	–	>0.05	<3	>0.9	<0.10	<0.08	<0.05	>0.9	>0.9	>0.9
Four-factor	385.748	146	<0.001	2.642	0.917	0.058	0.066	0.0466	0.947	0.918	0.938
One-factor	1,367.24	152	<0.001	9	0.72	0.128	0.15	0.099	0.731	0.708	0.697

A latent common-method variance (CMV) factor was also applied. The model converged successfully, yielding the following fit indices: *χ*^2^ = 250.633, df = 127, *p* < 0.001, *χ*^2^/df = 1.973, GFI = 0.947, RMSEA = 0.045, RMR = 0.045, CFI = 0.973, NFI = 0.947, NNFI = 0.963. All indices indicate good model fit. The CMV factor accounted for 7.2% of the total variance, below the 10% criterion commonly used to denote minimal common-method bias (Podsakoff et al., 2003).

Moreover, configural, metric, and scalar models were estimated for various groups of participants (total *N* = 486). For STEM (*n* = 236) versus non-STEM (*n* = 250) participants, the configural model exhibited excellent fit (CFI = 0.934, TLI = 0.923, RMSEA = 0.046); metric invariance was supported with ΔCFI = 0.002 and ΔRMSEA < 0.001. Full scalar constraints reduced CFI to 0.930 (ΔCFI = 0.004, ΔTLI = 0.004)—well below the 0.01 threshold, while RMSEA remained 0.045 (PCLOSE = 0.945), indicating close fit throughout. For male (*n* = 231) versus female (*n* = 255), the configural model exhibited excellent fit (CFI = 0.944, TLI = 0.934, RMSEA = 0.043); metric invariance was supported with ΔCFI = 0.003 and ΔRMSEA < 0.001. Full scalar constraints reduced CFI to 0.931 (ΔCFI = 0.013); after freeing the intercept of item Q19 (MI = 7.355), the partial scalar model achieved CFI = 0.934, ΔCFI = 0.010, and ΔTLI = 0.003, well below the 0.01 threshold, while RMSEA remained 0.044 with PCLOSE = 0.979. Thus, measurement invariance is further supported across gender (partial scalar) and disciplinary clusters (full scalar), demonstrating the EpiCT-CI scale’s applicability for cross-group comparisons.

**Figure 5:**
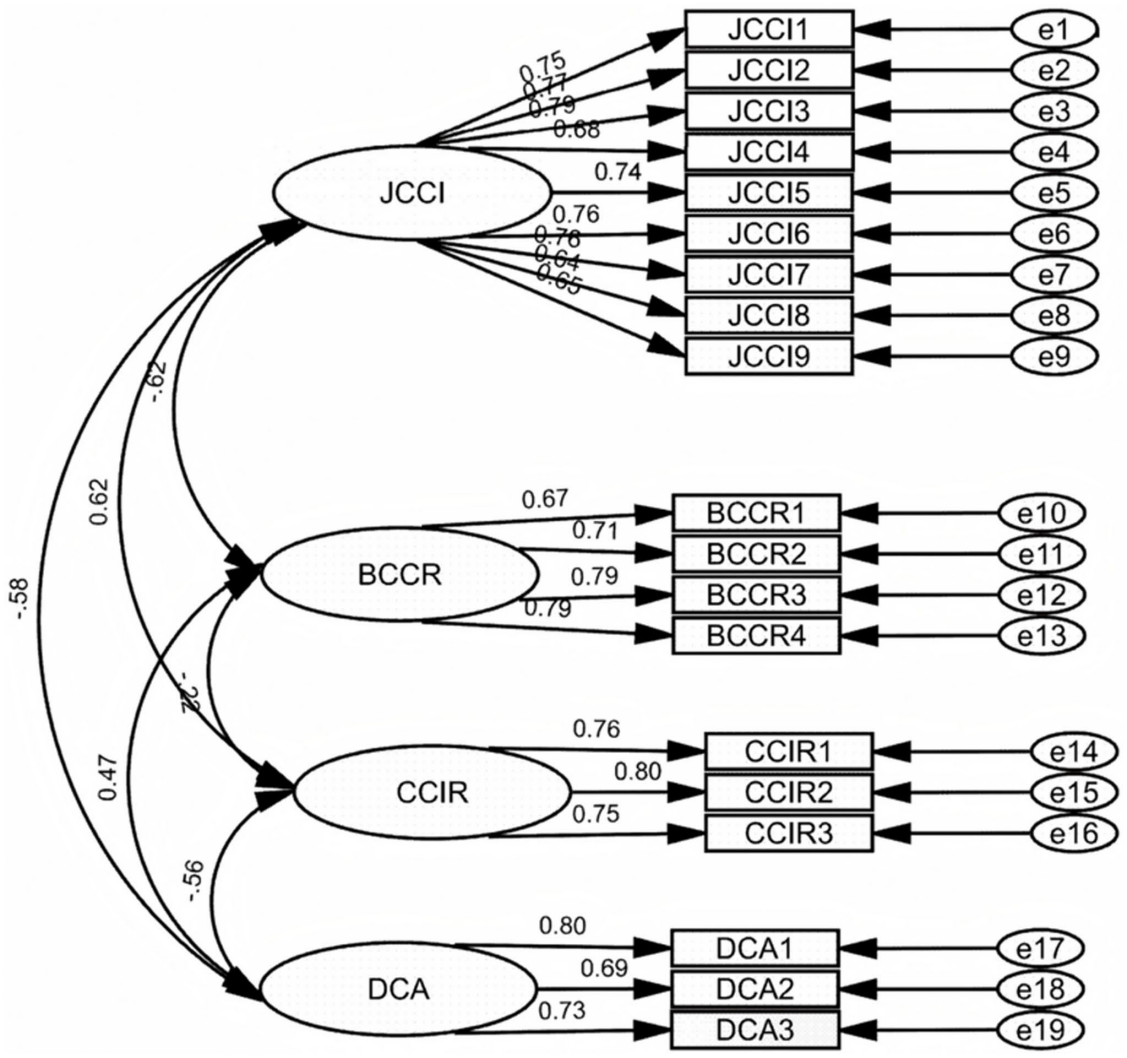
The model of CFA.

Figure 5 demonstrates the CFA model that illustrates the potential relationship between factors JCCI, BCCR, CCIR, and DCA. Brown (2015) states that factor correlations less than 0.8 indicate acceptable discriminant validity. The result in Figure 5 revealed that the latent factor correlations lie between −0.22 and 0.62, and standardized item loadings range from 0.64 to 0.8, supporting both convergent and discriminant validity. Among the four latent dimensions of the EpiCT-CI scale, the strongest positive association emerges between JCCI and CCIR (*r* = 0.62), indicating that respondents who enjoy exploring cultural issues tend to be more inquisitive about their cultural identity. In contrast, JCCI correlates negatively with both BCCR (*r* = −0.62) and DCA (*r* = −0.58), suggesting that the more joy respondents experience during inquiry, the less boredom they feel during reflection and the less distress they report while adapting to new cultural contexts. BCCR shows a moderate positive correlation with DCA (*r* = 0.47), implying that boredom during reflection coincides with heightened adaptation distress, and a modest negative correlation with CCIR (*r* = −0.22), implying that boredom may be associated with identity-focused curiosity. Finally, CCIR correlates negatively with DCA (*r* = −0.56), thus greater curiosity about cultural identity is linked to lower levels of adaptation distress. All six correlations are significantly below 0.8 (Brown, 2015), confirming that the four factors encapsulate related yet distinct constructs.

#### 4.2.4 Criterion-related validity

The EpiCT-CI Scale’s criterion-related validity is examined through the Need for Cognition Scale (NFC) (18 items, Cacioppo et al., 1984) and the dimension of Openness in the Big Five Inventory (10 items, John et al., 2008) (see Appendices A, B). The questionnaires were distributed online to 225 students from various disciplines. Cronbach’s Alpha was 0.938, indicating a high level of response consistency. Pearson correlation analysis was employed to assess the validity. Table 10 reveals that JCCI is positively related to CCIR (0.641), OPENNESS (0.599), and NFC (0.527), but negatively related to BCCR (−0.779) and DCA (−0.759), suggesting that individuals who find joy in critical cultural inquiry and cultural identity reflections are more likely to be open-minded and fond of discovery. BCCR and DCA are positively related to each other but negatively with JCCI, CCIR, OPENNESS (−0.454, −0.628), and NFC (−0.489, −0.461), indicating that higher levels of boredom and distress in cultural adaptation result in lower levels of joy, curiosity, and open-mindedness, which hinder the process of exploring cultural diversity and cultural identity development. Furthermore, discriminant validity was examined by AVE and Heterotrait-Monotrait Matrix (HTMT). All AVE values exceeded 0.48 and satisfied the Fornell–Larcker (1981) criterion (Fornell and Larcker, 1981), as the square root of each AVE was consistently greater than its highest correlation with any other construct. All HTMT ratios between the JCCI, BCCR, CCIR, DCA, OPENNESS, and NCF ranged from 0.65 to 0.75, well below the 0.85 threshold, indicating no problematic overlap (Henseler et al., 2015). The correlation coefficients are statistically significant at the 0.01 level. This robust correlation provides strong evidence supporting the EpiCT-CI Scale’s criterion-related validity.

**Table 10:** Criterion-related validity results (*n* = 225).

Variable	JCCI	BCCR	CCIR	DCA	OPENNESS
JCCI					
BCCR	−0.779**				
CCIR	0.641**	−0.508**			
DCA	−0.759**	0.594**	−0.691**		
OPENNESS	0.599**	−0.454**	0.685**	−0.628**	
NFC	0.527**	−0.489**	0.434**	−0.461**	0.435**

**p < 0.01.

## 5 Discussion and conclusion

The EpiCT-CI Scale fills the gap by integrating critical thinking and cultural identity into assessing epistemic emotions. It considers how epistemic emotions influence individuals to process cross-cultural information. Critical thinking is an important concept for decision making, but most measurements of critical thinking take it for granted that the decision-maker is hyper-rational. However, due to the multifaceted differences of individuals, the cognitive process functions with emotions together. If we shift our focus to economics, we can see that cultures and emotions play a crucial role in economic decision-making. Research conducted by Pertl et al. (2024), involving 70,000 participants across approximately 74 countries, demonstrates that specific cultural contexts influence the connection between emotions and decision-making processes. This indicates that cultural identity and critical thinking are significant for understanding the complexities of epistemic emotion in intercultural contexts. Furthermore, traditional instruments often fall short of accommodating the dynamic nature of specific emotional experiences. The self-report questionnaire for EES (Pekrun et al., 2017) significantly contributes to this field. Based on that, the EpiCT-CI Scale incorporates epistemic emotions into the specific context. It examines how emotions like curiosity drive deeper inquiry, how confusion indicates the need for perspective-taking, and how frustration may lead to defensive closure.

The development of the EpiCT-CI Scale is based on the findings of Studies 1 and 2. According to Creswell and Clark (2017), the QUAL→QUAN approach is suitable for exploring the “psychological phenomena that differ by culture,” which can produce findings that are difficult to obtain with a single method. Epistemic emotions are usually investigated after the epistemic activities by EES (Pekrun et al., 2017), such as science (Muis et al., 2015a,b) or mathematics (Muis et al., 2015a,b). However, research within education and intercultural studies seldom pays attention to the role of epistemic emotions in managing cultural issues. Furthermore, inventories for critical thinking and cultural identity often emphasize “what to do” more than “how you feel.” This research yields the measurement of epistemic emotions that can be more specific. The results of Studies 1 and 2 establish the range of epistemic emotion expressions when students apply critical thinking and deal with cultural identity constructions. The collected data in Study 1 are from students’ comments, judgments, and narration about critical thinking during COVID-19. COVID-19 is a macro angle to understand Chinese students’ views about coping with cultural dilemmas by applying critical thinking. The data of Study 2 are about the students’ emotional experiences of analyzing and writing about Western and Eastern friendship issues mentioned in the articles of intercultural expert Roger Baumgarte. Friendship is a micro angle to comprehend individuals’ cultural identity in interpersonal relationships (Peng, 2023). Additionally, epistemic activities cannot be limited to reading, and thus, Studies 1 and 2 illustrate possibilities for applying various kinds of learning activities, such as critical writing. By rooting item generation in specific cultural-critical episodes, the results of Studies 1 and 2 establish the basis of the EpiCT-CI Scale instrument that captures how individuals feel in epistemic activities, not merely how they remember feeling.

Besides, the results of Studies 1 and 2 are complementary in functioning to develop the EpiCT-CI Scale. By the automatic sentiment coding of NVivo 15.0, Figures 1, 3 demonstrate that each participant has a dynamic map that comprises mixed, neutral, positive, and negative emotions. This result indicates epistemic emotions blend neutral, positive, and negative states rather than linear or valence simple progressions. Individuals seek to validate their identities through information processing (Trevors et al., 2016). This creates a complex learning environment, where information confirming or challenging one’s identity can evoke positive or negative epistemic emotions. Therefore, developing a more nuanced inventory that reflects epistemic emotions when individuals actively apply critical thinking in cultural identity constructions is important. Tables 1–4 show the different epistemic activities in applying critical thinking and cultural identity constructions. The results of Study 1 generated 30 items that encompass various epistemic emotions in coping with cultural issues when applying critical thinking. However, the initial 30-item pilot scale derived only from Study 1 cannot yield clear and robust constructs in the EFA. Consequently, a second qualitative study (Study 2) was conducted. As Table 4 illustrates, the results of Study 2 generated 22 items. Table 6 shows that integrating the findings of Studies 1 and 2 has resulted in the stable four-factor EpiCT-CI Scale (33 items) with conceptual coherence and clarity. The result of EFA indicates that both Studies 1 and 2 contribute significantly to the scale.

The EpiCT-CI Scale reveals the epistemic emotions nature of “information-oriented appraisals (Muis et al., 2018). Naar (2025) suggests that emotions are defined as “appraisal,” indicating that various emotions interact with each other depending on the context. The results of EFA encompass four constructs: Joy in Critical Cultural Inquiry (JCCI), Boredom in Critical Cultural Reflection (BCCR), Curiosity in Cultural Identity Reflection (CCIR), and Distress in Cultural Adaptation (DCA), which identify the group of positive and negative epistemic emotions. Each construct represents the dynamic emotional experiences when individuals explore and reflect on their cultural identity. Table 6 shows that JCCI and CCIR include the positive epistemic emotions suggested by Muis et al. (2015b) (curiosity, interest, surprise, enjoyment) and those in EES (Pekrun et al., 2017) (excitement, joy, happiness). BCCR and DCA include the typical negative ones like anxiety, confusion, frustration, boredom (Muis et al., 2015b), and nervousness, fear, and worry (Pekrun et al., 2017). Additionally, Table 6 demonstrates some newly identified epistemic emotions (confidence, optimism, disappointment, shock, stress), which are still in the final version of the 19-item scale. Table 7 illustrates that the scale encompasses the positive (joy, confidence, interest, enjoyment, curiosity, desire, surprise, excitement, optimism) and negative epistemic emotions (boredom, fear, nervousness, disappointment, shock). Among these, optimism, confidence, and fear are closely associated with cultural factors (Van Hemert et al., 2007). The EpiCT-CI Scale broadens the connections between epistemic emotions, critical thinking, and cultural identity. By incorporating cultural factors into the study of epistemic emotions, this research provides a perspective that epistemic emotions do not simply accompany critical thinking but actively influence the ongoing interaction between cultural identities.

Furthermore, the EpiCT-CI scale sheds light on comprehending how epistemic emotion occurs through evaluating, assessing, and judging different cultural information in critical thinking application and cultural identity constructions. Epistemic emotions are academic emotions related to beliefs (Nakamura, 2023), but can also occur in broader contexts. Muis et al. (2015a, 2015b, 2018), Pekrun (2006, 2024), and Pekrun et al. (2017) already provide a comprehensive framework for exploring epistemic emotion. Based on that, the EpiCT-CI Scale bridges emotions, cognitive ability, and cultures. Cultures significantly influence emotions, leading to diverse responses to the same information. The identity construction is a procedure of overcoming the challenges that entail selecting from among unlimited possibilities and constructing from the elements chosen “something deemed to be of value (Waterman, 1984).” According to Muis et al. (2018), if perceived control of the information is low but value is high, learners may experience anxiety. Conversely, if both control and value of information are high, learners are more likely to experience joy. If both are low, individuals are more likely to experience boredom. Therefore, the ability to evaluate, understand, and assess the “high” or “low” value of information is significant for epistemic emotion. In Chinese cultures, *The Book of Rites*, a Confucian classic, defines that “Emotion is the manifestation of human nature in motion (*情者, 性之动也*).” This indicates that emotions are never static, but a living, kinetic concept ceaselessly shaped and re-interpreted by external and internal elements. By incorporating critical thinking skills, the EpiCT-CI scale examines how individuals engage with information, reflect on their beliefs, and navigate cultural contexts, ultimately contributing to a deeper understanding of epistemic emotions’ “appraisal” nature.

## 6 Limitation

This research has some limitations that should be mentioned to guide future research in this field. First, some of our participants were undergraduate students in an arts college, while others were from different subjects. These findings may not apply to other populations. Future research should validate the EpiCT-CI scale in postgraduate students or professionals who work in intercultural contexts, such as international school educators or multicultural team leaders. Second, the learning activities range from traditional classroom settings to online formats. This study’s qualitative analysis of epistemic emotions only focuses on reading and writing. However, as an academic emotion, the analysis of epistemic emotions can be more diverse, such as the discussion, presentation, or interaction online. Third, future research should validate the EpiCT-CI Scale across various cultures, as responses may differ for each item due to cultural differences. Moreover, epistemic emotions are influenced by various social and psychological factors, making them a crucial aspect of human learning. Future research should focus more on the social, cultural, and psychological aspects of the interplay between epistemic emotions, cognitive ability, and identity, which gives us a dynamic understanding of how humans evaluate and comprehend the world.

## Data Availability

The original contributions presented in the study are included in the article/Supplementary material, further inquiries can be directed to the corresponding author.
