# Supplementary material for: When emotion meets reason: the development and validation of EpiCT-CI scale to measure epistemic emotions in critical thinking application and cultural identity constructions
Source: Front Psychol. 2025 Oct 15;16:1687003. doi: 10.3389/fpsyg.2025.1687003 (PMC12568516; doi:10.3389/fpsyg.2025.1687003)
Supplement: Supplementary file 1 [file Table_1.docx]

**Appendix A** The Need for Cognition Scale (NFC) (18 items, Cacioppo et al., 1984)

|  | Items |
| --- | --- |
| 1 | I prefer complex to simple problems. |
| 2 | I like to have the responsibility of handling a situation that requires a lot of thinking. |
| 3 | Thinking is not my idea of fun. (R) |
| 4 | I would rather do something that requires little thought than something that is sure to challenge my thinking abilities. (R) |
| 5 | I try to anticipate and avoid situations where there is a likely chance I will have to think in depth about something. (R) |
| 6 | I find satisfaction in deliberating hard and for long hours. |
| 7 | I only think as hard as I have to. (R) |
| 8 | I prefer to think about small daily projects to long term ones. (R) |
| 9 | I like tasks that require little thought once I’ve learned them. (R) |
| 10 | The idea of relying on thought to make my way to the top appeals to me. |
| 11 | I really enjoy a task that involves coming up with new solutions to problems. |
| 12 | Learning new ways to think doesn’t excite me very much. (R) |
| 13 | I prefer my life to be filled with puzzles I must solve. |
| 14 | The notion of thinking abstractly is appealing to me. |
| 15 | I would prefer a task that is intellectual, difficult, and important to one that is somewhat important but does not require much thought. |
| 16 | I feel relief rather than satisfaction after completing a task that requires a lot of mental effort. (R) |
| 17 | It’s enough for me that something gets the job done; I don’t care how or why it works. (R) |
| 18 | I usually end up deliberating about issues even when they do not affect me personally. |

(R)=reversed item
