# Supplementary material for: When emotion meets reason: the development and validation of EpiCT-CI scale to measure epistemic emotions in critical thinking application and cultural identity constructions
Source: Front Psychol. 2025 Oct 15;16:1687003. doi: 10.3389/fpsyg.2025.1687003 (PMC12568516; doi:10.3389/fpsyg.2025.1687003)
Supplement: Supplementary file 2 [file Table_2.docx]

**Appendix B** The dimension of Openness in the Big Five Inventory (10 items, John et al., 2008)

|  | Items |
| --- | --- |
| 1 | I am original, and I come up with new ideas. |
| 2 | I am curious about many different things. |
| 3 | I am ingenious and a deep thinker. |
| 4 | I have an active imagination. |
| 5 | I am inventive. |
| 6 | I am someone who values artistic and aesthetic experiences. |
| 7 | I am a person who prefers work that is routine. (R) |
| 8 | I am reflective and like to play with ideas. |
| 9 | I am someone who has few artistic interests. (R) |
| 10 | I am sophisticated in art, music, or literature. |

(R)=reversed item
